# Dashboard of Sentiment in Austrian Social Media During COVID-19

**DOI:** 10.3389/fdata.2020.00032

**Published:** 2020-10-26

**Authors:** Max Pellert, Jana Lasser, Hannah Metzler, David Garcia

**Affiliations:** ^1^Complexity Science Hub Vienna, Vienna, Austria; ^2^Section for Science of Complex Systems, Center for Medical Statistics, Informatics and Intelligent Systems, Medical University of Vienna, Vienna, Austria; ^3^Institute for Globally Distributed Open Research and Education, Stockholm, Sweden

**Keywords:** COVID-19, collective emotions, real-time monitoring, social media, digital traces, webscraping, dashboard, affective sciences

## Abstract

To track online emotional expressions on social media platforms close to real-time during the COVID-19 pandemic, we built a self-updating monitor of emotion dynamics using digital traces from three different data sources in Austria. This allows decision makers and the interested public to assess dynamics of sentiment online during the pandemic. We used web scraping and API access to retrieve data from the news platform derstandard.at, Twitter, and a chat platform for students. We documented the technical details of our workflow to provide materials for other researchers interested in building a similar tool for different contexts. Automated text analysis allowed us to highlight changes of language use during COVID-19 in comparison to a neutral baseline. We used special word clouds to visualize that overall difference. Longitudinally, our time series showed spikes in anxiety that can be linked to several events and media reporting. Additionally, we found a marked decrease in anger. The changes lasted for remarkably long periods of time (up to 12 weeks). We have also discussed these and more patterns and connect them to the emergence of collective emotions. The interactive dashboard showcasing our data is available online at http://www.mpellert.at/covid19_monitor_austria/. Our work is part of a web archive of resources on COVID-19 collected by the Austrian National Library.

## 1. Introduction

In 2020, the outbreak of COVID-19 in Europe lead to a variety of countermeasures aiming to limit the spread of the disease. These include temporary lockdowns, the closing of kindergartens, schools, shops, and restaurants, the requirement to wear masks in public and restrictions on personal contact. Health infrastructure was re-allocated with the goal of providing additional resources to tackle the emerging health crisis triggered by COVID-19. Such large-scale disruptions of private and public life can have tremendous influence on the emotional experiences of a population.

Governments must build on the compliance of their citizens with these measures. Forcing the population to comply by instituting harsh penalties is not sustainable in the longer run, especially in developed countries with established democratic institutions like in most of Europe. On the scale of whole nations, very strict policing also faces technical limits and diverts resources from other duties. In addition, recent research shows that, when compared to enforcement, the recommendation of measures can be a better motivator for compliance (Del Fava et al., 2020). Non-intrusive monitoring of emotional expressions of online populations enables to identify problems early on, with the hope to provide the means to resolve them.

Due to the rapid development of the response to COVID-19, it is desirable to produce up-to-date observations of public sentiment, especially when restrictive countermeasures are activated. At the same time, it is hard to quantify sentiment at large scales and high temporal resolution. Policy decisions are usually accompanied by representative surveys of public sentiment that, however, suffer from several shortcomings. First, surveys depend on explicit self-reports which do not necessarily align with actual behavior (Baumeister et al., 2007). In addition, conducting surveys among larger numbers of people is time consuming and expensive. Lastly, a survey is always just a snapshot of public sentiment at a single point in time. Often, by the time a questionnaire is constructed and the survey has been conducted, circumstances have changed and the results of the survey are only partially valid.

Online communities are a complementary data source to surveys when studying current and constantly evolving events. Their digital traces reveal collective emotional dynamics almost in real time. We gathered these data in the form of text from platforms such as Twitter and news forums (derstandard.at and a student platform) where large groups of users discuss timely issues. We observed a lot of activity online, and we found clear increases during the nation-wide lock down of public life. For example, our data shows Austrian Twitter saw a 73% increase in posts from 9 March 2020 compared to before (2019-01-01 until 2020-03-08). Livetickers at news platforms are a popular format that provides small pieces of very up-to-date news constantly over the course of a day. This triggers fast posting activity in the adjunct forum. By collecting these data in regular intervals, we faced very little delay in data gathering and analysis and provide a complement to survey-based methods. Our setup has the advantage of bearing low cost while featuring a very large sample size. The disadvantages include more noise in the signal due to our use of automated text analysis methods, such as sentiment analysis. Additionally, if only information from one platform is considered, this might result in sampling a less representative part of the population than in surveys where participant demographics are controlled. This population bias can be mitigated by selecting data sources that are diverse in terms of specific demographic attributes (like age or political affiliation). However, less liberal online newspaper forums may be not active enough to allow for large-scale web scraping of postings (krone.at, OE24.at). Also, corporate decisions to stop providing API access (Facebook) or privacy preserving features like end-to-end encryption (Whatsapp) pose technical and ethical limits to this goal. Systematic approaches to account for account for these and other errors and biases at different stages of research have been adapted to digital traces data (Olteanu et al., 2019; Sen et al., 2019).

We showcase the monitoring of social media sentiment during the COVID-19 pandemic for the case of Austria. The developments around COVID-19 in Austria have been closely followed by the rest of Europe. As the virus started spreading in Europe on a larger scale in February 2020, stringent measures were implemented comparatively early in Austria (Desvars-Larrive et al., 2020). Using data from Austria allowed us to build a quite extensive, longitudinal account of first-hand discussions on COVID-19. Additionally, Austria's political system and its public health system have all the capacities of a developed nation to tackle a health crisis (kurier.at, 2020). We therefore expect the users of online platforms in Austria to express the personal, emotional reaction to the event without being overwhelmed by lack of resources and resulting basic issues of survival.

Interactive online dashboards are an accessible way to summarize complex information to the public. During COVID-19, popular dashboards have conveyed information about the evolution of the number of COVID-19 cases in different regions of Austria (Austrian Ministry for Health, 2020) and globally (CSSE, 2020). Other dashboards track valuable information such as world-wide COVID-19 registry studies (Thorlund et al., 2020). Developers of dashboards include official governmental entities like the national ministry of health as well as academic institutions and individual citizens. Many of these dashboards display raw data together with descriptive statistics of “hard” facts and numbers on COVID-19. Other researchers have used sentiment analysis to study patterns of sentiment dynamics during COVID-19 (Lwin et al., 2020). We aimed to combine these approaches by developing a dashboard showcasing processed data from three different sources to track the sentiment in Austrian social media during COVID-19. It is easily accessible online and updated on a daily basis to give feedback to authorities and the interested general public.

## 2. Method

**Figure 1:**
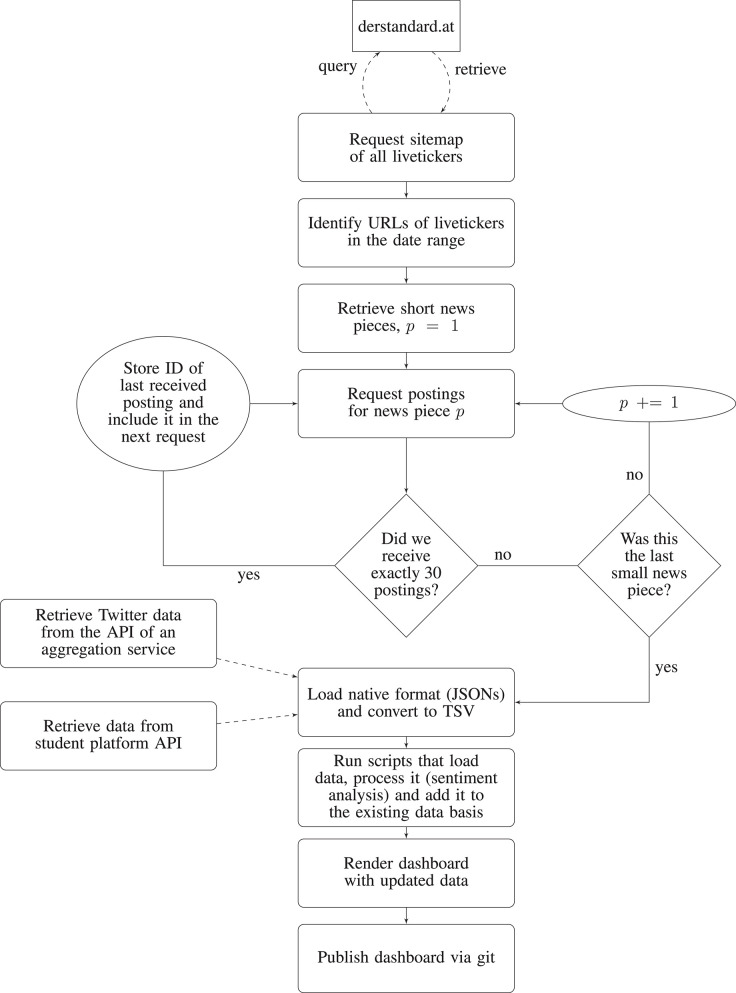
Flowchart of the daily routine of updating the dashboard. We run this routine as a cronjob each day in the morning at 7 a.m.

We retrieve data from three different sites: a news platform, Twitter, and a chat platform for students. All data for this article was gathered in compliance with the terms and conditions of the platforms involved. Twitter data was accessed through Crimson Hexagon (Brandwatch), an official Twitter partner. We used Crimson Hexagon for two reasons. First, it allows accessing the full history of Twitter, in comparison to a sample of only 1% accessible via the free Twitter API. As previous research has shown, this sample is potentially biased and not a fully random, representative part of Twitter activity (Pfeffer et al., 2018). Second, Crimson Hexagon's algorithm provides a reliable estimate of users' location, by taking into account the entire information available in the profile of a user (text in user bio, tweet text etc.). This procedure is more reliable than sampling geolocated users through the Twitter API and gives a larger sample of users in Austria. The platform for students and derstandard.at gave us their permission to retrieve the data automatically from their systems. A daily recurring task is set up on a server to retrieve and process the data and to publish the updated page online (for a description of the workflow see Figure 1).

The news platform derstandard.at was an internet pioneer as it was the first German language newspaper to go online in 1995. From February 1999, it started entertaining an active community, first in a chatroom (derstandard.at, 2018). In 2008, the chatroom was converted to a forum that is still active today and allows for posting beneath articles. Users have to register to post and they can up- and down-vote single posts. In 2013, a platform change made voting more transparent by showing which user voted both positive and negative. According to a recent poll (derstandard.at, 2020a), derstandard.at is considered both the most trustful and most useful source of information on COVID-19 in Austria. Visitors come from Austria but also from other parts of the German-speaking area. In 2020, derstandard.at was visited by 2,546,000 unique users per month that stay on average 06:42 min on the site and request a total of 215,974,000 subpages (derstandard.at Sales Team, 2020). To cover the developments around COVID-19, daily livetickers (except Sundays) were set up on derstandard.at. Supplementary Figure 1 shows an example of the web interface of such a liveticker.

As no dedicated API exists for data retrieval from derstandard.at, we used web-scraping to retrieve the data (under permission from the site). First, we requested a sitemap and identified the relevant URLs of livetickers. Second, we queried each small news item of each of the livetickers. We received data in JSON format and flattened and transformed the JSON object to extract the ID of each small news piece. Third, we queried the posts attached to that ID in batches. This was necessary because derstandard.at does not display all the posts at once beneath a small news item. Instead, the page loads a new batch of posts as soon as the user reaches the bottom of the screen. This strategy was chosen to not overcrowd the interface, as the maximum number of posts beneath one small news item can be very high (up to 2,293 posts in our data set). By following our iterative workflow to request posts, we could circumvent issues of pagination. Finally, after we received all posts, we transformed the JSON objects to tabulator-separated value files for further analysis. This approach is summarized in the upper part of Figure 1.

To retrieve daily values for our indicators from Twitter, we relied on the Forsight platform by Crimson Hexagon, an aggregation service of data from various platforms, including Twitter. Twitter has an idiosyncratic user base in Austria, mainly composed of opinion makers, like journalists and politicians. In the case of studying responses to a pandemic, studying these populations gives us an insight into public sentiment due to their influence in public opinion. Yet, one should keep in mind that Twitter users are younger, more liberal and have higher formal education than the general population (Pew Research Center, 2019). This is similar for users of derstandard.at (derstandard.at Sales Team, 2020).

As a third and last source, we included a discussion platform for young adults in Austria^1^. The discussions on the platform are organized in channels based on locality, with an average of 580±390 (mean ± standard deviation) posts per day from 2020-01-01 to 2020-05-27. The typical number of posts per day on the platform dropped from 830±260 (January–April) to 160±80 (April–May). This drop occurred due to the removal of the possibility to post anonymously on April 4th 2020 in order to prevent hate speech. Based on data from this platform, we studied the reaction of the special community of young adults in different Austrian locations, with the majority of posts originating in Vienna (9%), Graz (8%), and other locations (83%).

To assess expressions of emotions and social processes, we matched text in posts on all three platforms to word classes in the German version of the Linguistic Inquiry and Word Count (LIWC) Dictionary (Wolf et al., 2008), including anxiety, anger, sadness, positive emotions, and social terms. LIWC is a standard methodology in psychology for text analysis that includes validated lexica in German. It has been shown that LIWC, despite its simplicity, has an accuracy to classify emotions in tweets that is comparable to other state of the art tools in sentiment analysis benchmarks (Ribeiro et al., 2016). Previous research has shown that LIWC, when applied to large-scale aggregates of tweets, has similar correlations with well-being measures as other, more advanced text analysis techniques (Quercia et al., 2012; Jaidka et al., 2020). Since within the scope of this study only text aggregates were be analyzed, LIWC is an appropriate method and can be applied to all sorts of text data that is collected for the monitor. For the prosocial lexicon, we translated a list of prosocial terms used in previous research (Frimer et al., 2014), including for example words related to helping, empathy, cooperating, sharing, volunteering, and donating.

We adapt the dictionaries to the task at hand by excluding most obvious terms that can bias the analysis, as done in recent research validating Twitter word frequency data (Jaidka et al., 2020). Specifically, we cleaned the lists for (1) words that are likely more frequently used during the COVID-19 pandemic, e.g., by news media and do not necessarily express an emotion (sadness: tot*; anger: toete*, töt*, töte*; positive: heilte, geheilt, heilt, heilte*, heilung; prosocial: Heilverfahren, Behandlung, Behandlungen, Dienstpflicht, Öffentlicher Dienst, and Digitale Dienste all matching Dienst*), (2) potential mismatches unrelated to the respective emotion (sadness: Harmonie/harmlos matching Harm*; positive: äußerst; prosocial: Dienstag matching Dienst*), (3) specific Austria-related terms like city names (sadness: Klagenfurt matching klagen*) or events (sadness: Misstrauensantrag matching miss*), and (4) Twitter-related terms for the analysis of Tweets only (prosocial: teilen, teilt mit).

For text from derstandard.at, we averaged the frequency of terms per post to take into account the varying lengths of posts. As Twitter has a strict character limit of 280 characters per post, Crimson Hexagon provides the number of tweets containing at least one of the terms, and we then used this to calculate the proportion of such posts. Posts have a median length of 61 characters in derstandard.at, 101 characters in Twitter, and 51 characters in the chat platform for young adults. In our analysis, we needed to exclude periodic weekday effects, as for example people express higher positive affect on weekends (Golder and Macy, 2011). To correct for this, we established a weekday baseline of our indicators. Then, we computed the relative difference of each post to the baseline values of the corresponding weekday. For derstandard.at data, the weekday baselines were computed from all posts to derstandard.at articles in the year 2019. We used the main website articles for this instead of livetickers because during 2019, livetickers were mainly used to cover sport events (for an example see https://www.derstandard.at/jetzt/livebericht/2000088339740/bundesliga-live-lask-sturm) or high-profile court cases (https://www.derstandard.at/jetzt/livebericht/2000088169126/buwog-prozess-vermoegensverwalter-stinksauer-auf-meischberger). Thereby, we chose a slightly different medium for our baselines to avoid having a topic bias in the baselines. Nonetheless, the data came from the same platform with the same layout and functionalities and an overlapping user base: 14,422 users (75% of total unique users in the livetickers) in our data set that are active at livetickers also post normal articles. The speed of posting may differ slightly because the article is typically posted in a final format, whereas small news pieces are added constantly in livetickers. For the other data sources, we corrected by computing weekday baselines for the indicators from the start of period available to us (Twitter back to 2013-01-01, chat platform for young adults back to 2019-01-01) to January 2020.

Finally, we combined the processed data and render an interactive website. For this, we used “plotly” (Sievert, 2018), “rflexdashboard” (Iannone et al., 2018), and “wordcloud2” (Lang, 2020) in R (R Core Team, 2019), and the “git” protocol to upload the resulting HTML page to GitHub Pages. Using versioning control allowed us to easily revert the page to a previous state in case of an error.

## 3. Results

We tracked the expressed sentiment on social media platforms in Austria during COVID-19 and made our findings available as an interactive online dashboard that is updated daily. We displayed the time series almost in real-time with a small delay to catch all available data (see **Figure 3** using derstandard.at as a data source). It has features such as the option to display the number of observations by hovering over the data point or to isolate lines and to compare only a subset of indicators. The dashboard can be accessed online at http://www.mpellert.at/covid19_monitor_austria/.

Table 1 shows several descriptive statistics of the data sets used. For derstandard.at, we retrieved 111 livetickers with 10,013 small news items. On average, users publish 183±156 posts under each of those items in the time period of interest (2020-03-09 to 2020-06-03). Posts have a median length of 61 characters (see Supplementary Figure 2 for a histogram of the length of posts). Posts provide immediate reactions by the users of derstandard.at: The median is at 24.7 s for the first post to appear below a small news item.

**Table 1:** Descriptive statistics showing relevant aspects of the data sources.

	**derstandard.at**	**Twitter**	**Student platform**
# Posts	1,827,576	2,001,420	158,022
Mean # Posts per day	21,007	23,005	1,816
# Unique users	19,263	594,500	NA
Fraction posemo	0.333	0.435	0.383
Fraction anxiety	0.036	0.031	0.032
Fraction anger	0.062	0.057	0.062
Fraction sad	0.083	0.064	0.09
Fraction social	0.561	0.559	0.592
Fraction prosocial	0.105	0.191	0.099

*Numbers refer to the period from 9 March to 3 June 2020 (87 days). The total number of Twitter users in Austria in January 2019 is taken from the report of DataReportal (2019). Fractions refer to the number of posts containing at least one term from the relevant dictionary category in LIWC divided by the total number of posts*.

**Figure 2:**
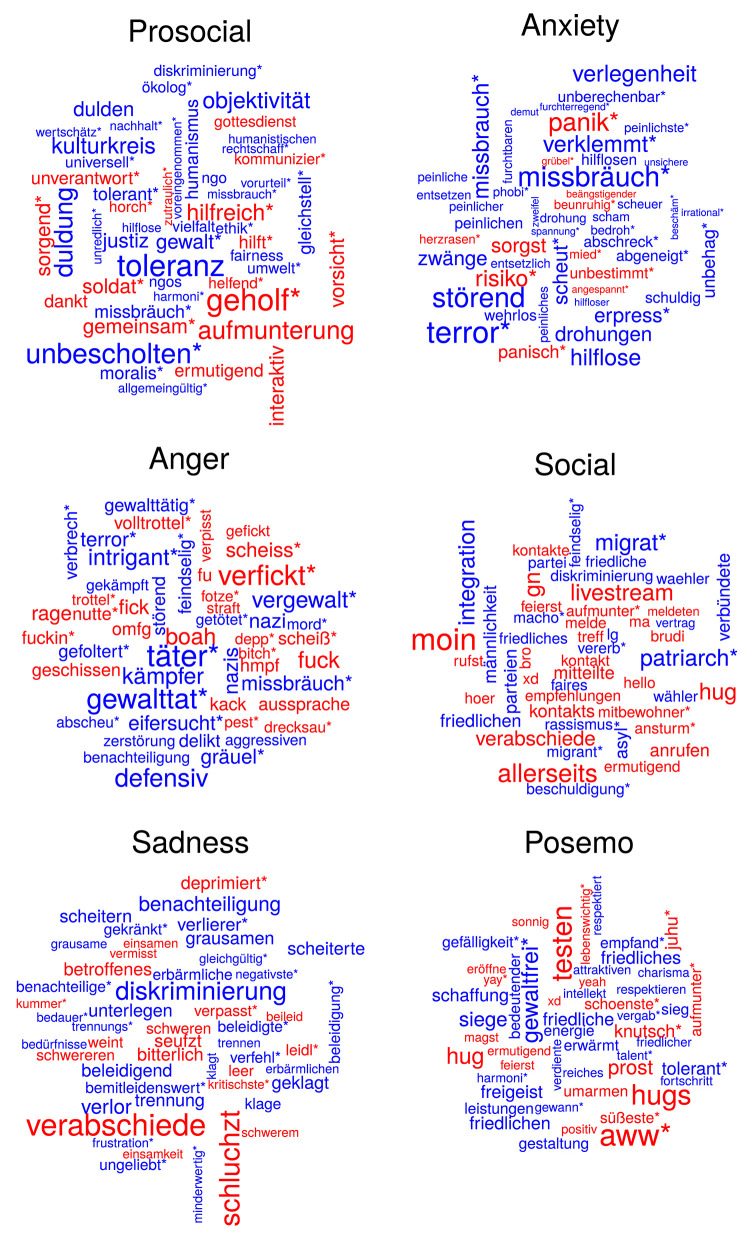
Wordclouds for posts on derstandard.at showing the matched words in each category. Size corresponds to the magnitude and color to the direction of change: blue and red mean less and more prevalent in the COVID-19 livetickers than in the normal articles of 2019, respectively. To be included, dictionary terms have to appear at least 10 times in both corpora.

In Figure 2, we use word clouds to provide an intuitive visualization (Felix et al., 2018) of the emotional content of posts. While livetickers on COVID-19 cover the period from 2020-03-09 until 2020-06-03, the baseline includes normal articles on derstandard.at from 2019. To highlight changes in language use during COVID-19, our word clouds compare word frequency in the livetickers with the baseline: the size of words in the clouds is proportional to $\left|\log\left(\frac{prob_{livetickers}}{prob_{baseline}}\right)\right|$, where *prob*_*baseline*_ and *prob*_*livetickers*_ refer to the frequency of the dictionary term compared to the frequency of all matches of terms in that category, in the baseline and the livetickers, respectively. Color of words corresponds to the sign of this quantity: red means positive, i.e., the frequency of the word increased in the livetickers, whereas blue signifies that the usage of the word decreased. By combining this information, our word clouds give an impression on how the composition of terms in the dictionary categories changed during COVID-19.

The dashboard illustrates early and strong increases in anxiety across all three analyzed platforms starting at the time of the first confirmed cases in Austria (end of February 2020). A first initial spike of anxiety terms occurs on all three platforms around the time the first positive cases were confirmed and news about the serious situation in Italy were broadcast in Austria. About 2 weeks later, levels rose again together with the number of confirmed cases, reaching particularly high levels in the week before the lock-down on 16 March. Afterwards, they gradually dropped again. In total, levels of anxiety expression did not return to the baseline for more than 6 weeks from 2020-02-22 to 2020-04-07 on Twitter. On derstandard.at, levels also remained above the baseline for more than 4 weeks in a row. Timelines for Twitter and derstandard.at also show a clear and enduring decrease of anger-related words starting in the week before the lock-down, as discussions of potentially controversial topics other than COVID-19 become scarcer. This decrease lasted for 4 weeks on derstandard.at (from 2020-02-21 to 2020-04-23) but was particularly stable on Twitter, where anger terms remained less frequent than in 2019 for 2.5 months in a row (from 2020-03-09 to 2020-05-29). In contrast, prosocial and social terms show opposing trends on these two platforms: they increased slightly but do so for more than 2 months on Twitter where people share not only news but also talk about their personal lives. In contrast, they decreased for more than 3 months in a row on derstandard.at, where people mostly discuss specific political events or topics. The increase of sadness-related expressions is smaller than changes in anxiety and anger but also lasted for about 1 month on Twitter and 2 weeks on derstandard.at. Interestingly, positive expressions were used slightly more frequently on all three platforms for long periods since the outbreak. This trend is visible from the beginning of March on the student platform and derstandard.at, and further increases since restrictions on people's lives have reduced. In total, positive expressions are more frequent than baseline during the last 2.5 months (as of 13th of June) on derstandard.at. An analysis of collective emotions in Reddit comments from users in eight US cities found results similar to ours, including spikes in anxiety and the decrease in anger (Ashokkumar, 2020), which suggests that some of our findings might generalize to other platforms and countries.

**Figure 3:**
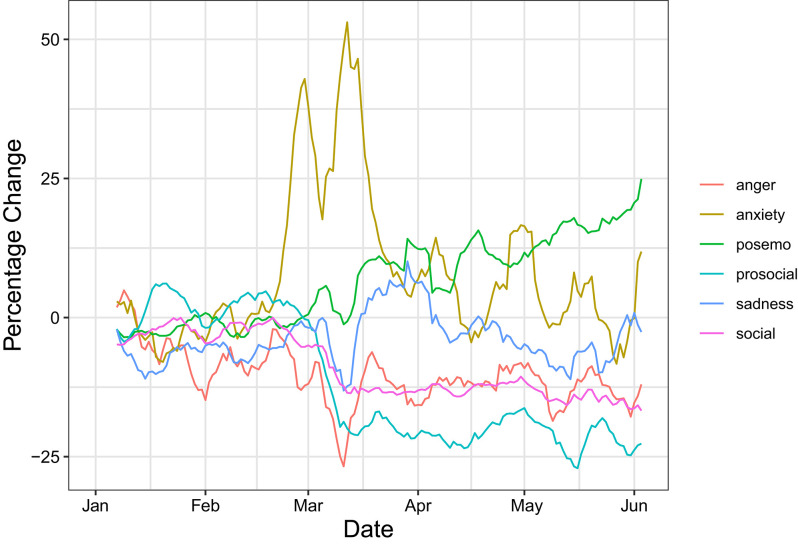
Timeline of the indicators for derstandard.at during the COVID-19 period. Values correspond to the percentage change against the baseline of the full year 2019. Lines are smoothed using a 7 day moving average. To be included, dictionary terms have to appear at least 10 times in both corpora.

We identified the following events in Austria corresponding to anxiety spikes in expressed emotions in social media. Unrelated to COVID-19, there was reporting on a terrorist attack in Hanau, Germany, on 2020-02-25. The first COVID-19 case in Austria was reported on 2020-02-25 and the first death on 2020-03-12. The first press conference, announcing bans of large public events and University closures as first measures, happened on 2020-03-10. It was followed by strict social distancing measures announced on 2020-03-15, starting on the day after. All COVID-19-related dates are derived not from the text data but are rather externally determined based on the most important events in Austria at the beginning of the outbreak. We then aimed to provide possible interpretations for spikes left unexplained by these landmark events. We found that the first spike (“terrorist attack in Hanau”) entirely vanishes if one excludes the word “terror,” which is included in the anxiety and anger word list. The overall patterns in the monitor of sentiment in Figure 3 show that Austrian user's expressions of anxiety increased, whereas anger decreased in our observation period.

Our dashboard analyses a part of public discourse. We assumed that the lockdown of public life increased tendencies of the people living in Austria to move debates online. Users that take part in these discussions often form very active communities that sometimes structure their whole day around their posting activities. This is reflected in our data in the word clouds of Figure 2 from the increased usage of greetings (category “social”), marking the start or the end of a day such as “moin”/“good morning” or “gn”/“good night.”

In an effort to provide an archive of Austrian web resources for future reference, the Austrian Nationals Library (ÖNB) monitors the dashboard and stores changes. There are a number of such initiatives also in other nations (Gomes et al., 2011) with the earliest and most famous example being archive.org. Through selective harvesting of resources connected to COVID-19, the dashboard is part of the ÖNB collection “Coronavirus 2020” (https://webarchiv.onb.ac.at/).

## 4. Discussion

Our results show patterns in the change of language use during COVID-19. In the anger category, words related to violence and crime are less frequent in livetickers since COVID-19 compared to 2019, indicating that reports and discussions about violent events, or possibly even these events themselves, become less frequent as the public discourse focuses on events related to the pandemic. For anxiety, the most remarkable change is a reduction in words related to terror and abuse, accompanied by a smaller increase of terms linked to panic, risk, and uncertainty. In the sadness category, the verb “verabschiede”/“saying goodbye” appears almost nine times more often in the livetickers. For prosocial words, terms referring to helping, community and encouragement increased. From the social terms, the word “empfehlungen”/“recommendations” occurs slightly more frequently, while topics of migration, integration, and patriarchy are less often discussed. Finally, positive terms that increase the most are the expression of admiration “aww*” and “hugs,” indicating that people send each other virtual hugs instead of physical ones.

The sentiment dynamics on social media platforms can be influenced by content that spreads fear and other negative emotions. Timely online emotion monitoring could help to quickly find traces of such campaigns by actors, that have an interested in sabotaging communication efforts. But legitimately elected governments with arguably different intentions can also follow the controversial strategy of steering emotions to alert the population to the danger of a threat. For example, democratically elected actors can deliberately elicit emotions such as fear or anxiety to increase compliance from the top down. Such a strategy has reportedly been followed in Austria (orf.at, 2020) and other countries like Germany (abgeordnetenwatch.de, 2020). Reports about the deliberate stirring of fear by the Austrian government are reflected in a spike of anxiety on 2020-04-27 in Figure 3. This spike reduced by one third (from 1,212 to 806 tweets) when tweets containing one of the following words were excluded: kurz, kanzler, schüren, angstmache, angstmacherei panikmache, protokoll, lügen, bewusst, strategie, kanzler, regierung, politik, bevölkerung, and bürger. This suggests it is partially associated to media reports about the Austrian government strategically stirring anxiety to raise awareness about COVID-19. The spikes of anxiety at the beginning of March in the early stages of the COVID-19 outbreak may also have been reinforced by these anxiety eliciting strategies.

Emotion dynamics are different in crisis times. Individual emotional expressions decay very fast (Pellert et al., 2020). Typically, collective emotions last longer but also return to the baseline within days even after catastrophic events like natural disasters or terrorist attacks (Gruebner et al., 2017; Garcia and Rimé, 2019). In contrast, changes during the COVID-19 pandemic in Austria have lasted several weeks for most analyzed categories (up to 12 weeks in some cases). Different to one-off events, threat from a disease like COVID-19 is more diffuse, and the emotion-eliciting events are distributed in time. In addition, measures that strongly affect people's daily lives over a long period of time, as well as high level of uncertainty, likely contribute to the unprecedented changes of collective emotional expression in online social media.

The dashboard gives opinion makers and the interested public a way to observe collective sentiment vis-a-vis the crisis response in the context of a pandemic. It has gained attention from Austrian media (APA, 2020), and from the COVID19 Future Operations Clearing Board (Federal Chancellery, Republic of Austria, 2020), an interdisciplinary platform for exchange and collaboration between researchers put in place by the Federal Chancellery of the Republic of Austria. Especially during the first weeks of the crisis, multiple newspapers reported on the changes of emotional expressions in online platforms (derstandard.at, 2020b; Ennemoser, 2020; Keymedia Wien, 2020; Science.ORF.at, 2020; Wiener Zeitung Online, 2020). Timely knowledge about collective online emotional expressions is valuable for evaluating risk-communication as well as for improving the preparedness and efficiency of emergency services during a pandemic.

## Data Availability Statement

The raw data supporting the conclusions of this article can be downloaded from the online version of the dashboard.

## Author Contributions

MP and DG designed the research. MP retrieved derstandard.at data, processed and analyzed all data, and implemented the dashboard. JL retrieved data for the platform for young adults. HM retrieved data for Twitter, wrote down the methods, and created the result reports for the dashboard. MP, JL, and HM wrote the draft of the manuscript. All authors provided input for writing and approved the final manuscript.

## Conflict of Interest

The authors declare that the research was conducted in the absence of any commercial or financial relationships that could be construed as a potential conflict of interest.

## Footnotes

^1^Since the platform owners agreed to share data within the scope of the ongoing COVID-19 crisis but do not want to be named in public, we refrained from giving details that could identify the platform. However, it must be noted that all data analyzed here was publicly available and we did not analyze any private digital traces.
