# Supplementary material for: Dashboard of Sentiment in Austrian Social Media During COVID-19
Source: Front Big Data. 2020 Oct 26;3:32. doi: 10.3389/fdata.2020.00032 (PMC7931924; doi:10.3389/fdata.2020.00032)
Supplement: Supplementary file 1 [file Presentation_1.PDF]

frontiers

Supplementary Material

1 DERSTANDARD.AT INTERFACE

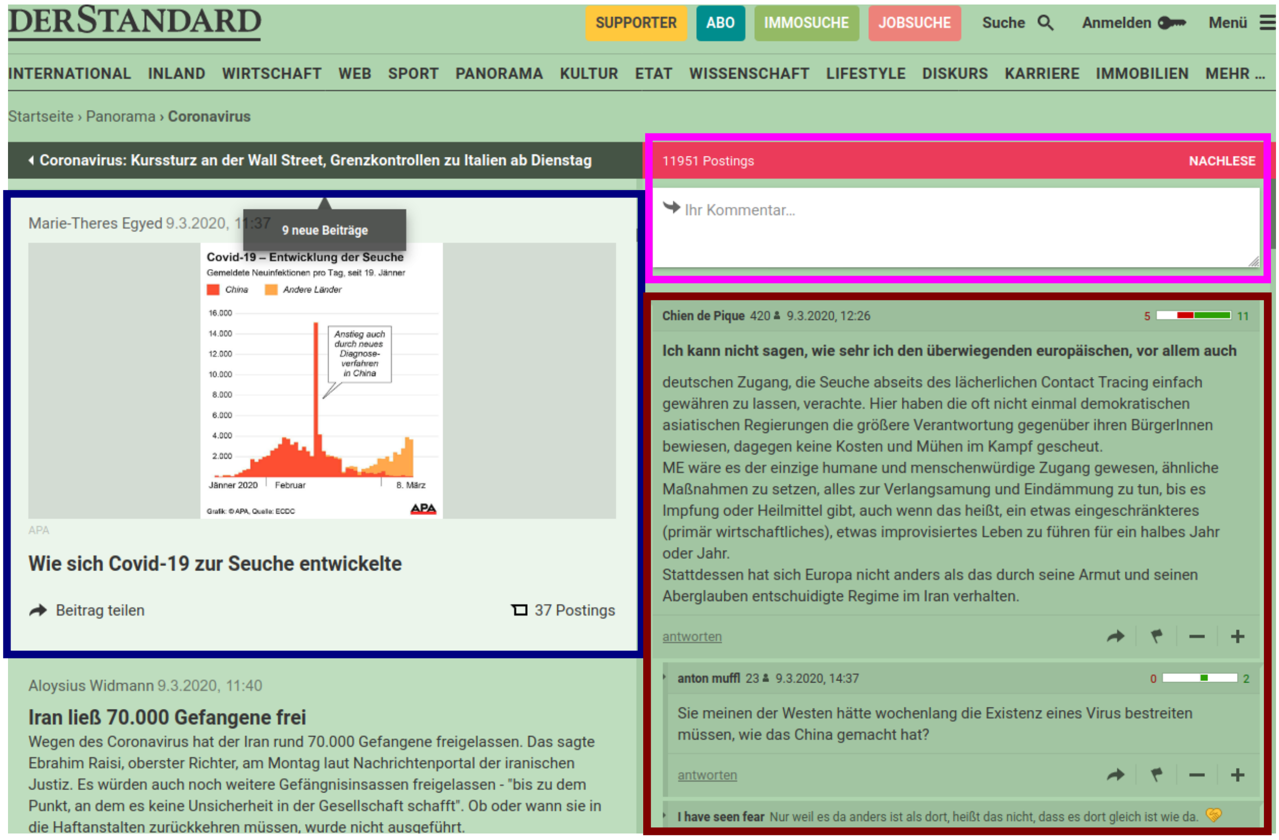

Figure S1. Example of derstandard.at liveticker The rectangle in navy blue shows one small news item. New postings can be added using the form to the right of it (fuchsia rectangle) and the current total number of postings in the liveticker is shown. Below, the postings connected to the news item are displayed (maroon rectangle).

2 LENGTH OF POSTINGS AT DERSTANDARD.AT

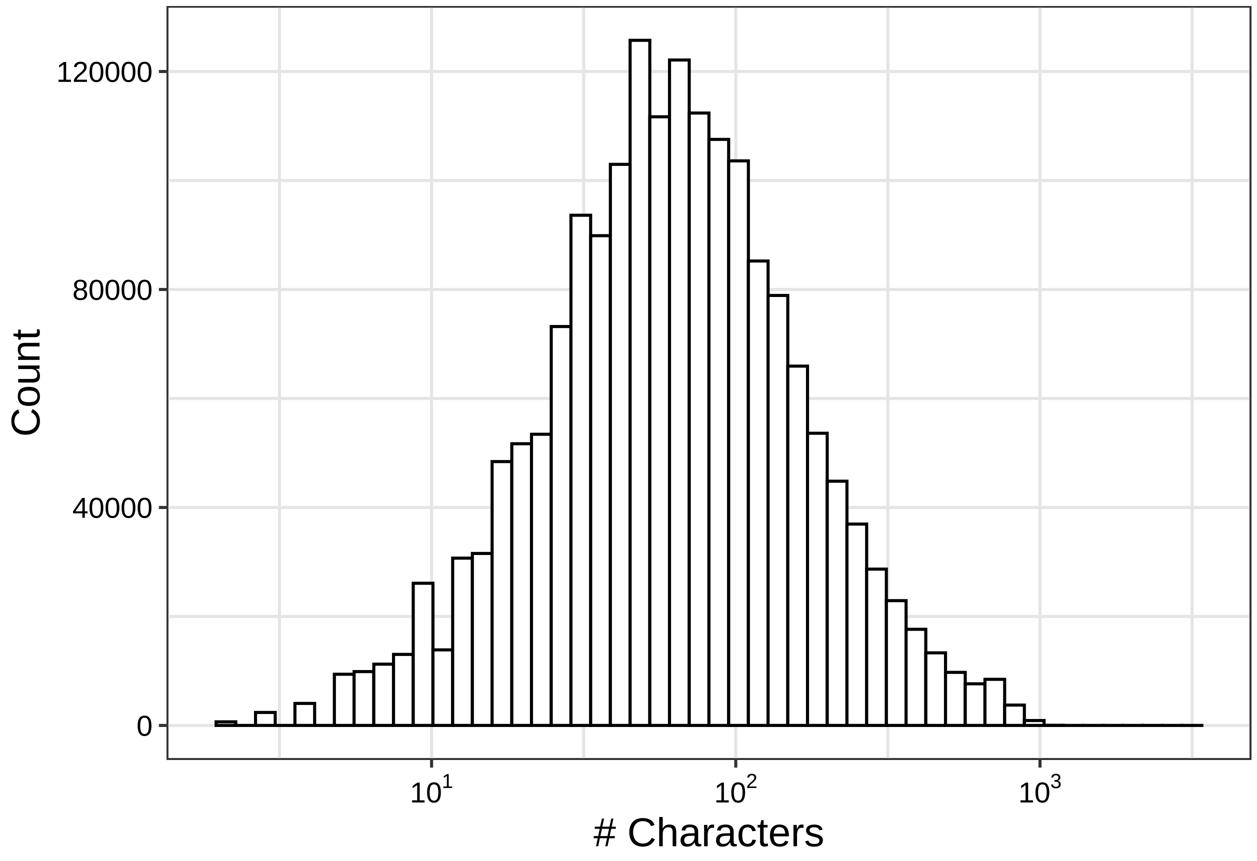

Figure S2. Histogram of the number of characters in derstandard.at postings with a log-transformed x-axis.
